# Assessing Transformations of Algal Organic Matter in the Long-Term: Impacts of Humification-Like Processes

**DOI:** 10.3390/ijms160818096

**Published:** 2015-08-05

**Authors:** Maud Leloup, Virginie Pallier, Rudy Nicolau, Geneviève Feuillade-Cathalifaud

**Affiliations:** EA 4330 Groupement de Recherche Eau, Sol, Environnement (GRESE), Ecole Nationale Supérieure d'Ingénieurs de Limoges (ENSIL), University of Limoges, Parc d’ESTER Technopôle, 16 rue Atlantis, Limoges Cedex F-87068, France; E-Mails: virginie.pallier@ensil.unilim.fr (V.P.); rudy.nicolau@ensil.unilim.fr (R.N.)

**Keywords:** algae, cyanobacteria, transformation processes, organic matter, size fractionation, XAD fractionation

## Abstract

Algae and cyanobacteria are important contributors to the natural organic matter (NOM) of eutrophic water resources. The objective of this work is to increase knowledge on the modifications of algal organic matter (AOM) properties in the long term to anticipate blooms footprint in such aquatic environments. The production of AOM from an alga (*Euglena gracilis*) and a cyanobacteria (*Microcystis aeruginosa*) was followed up and characterized during the stationary phase and after one year and four months of cultivation, in batch experiments. Specific UV absorbance (SUVA) index, organic matter fractionation according to hydrophobicity and apparent molecular weight were combined to assess the evolution of AOM. A comparison between humic substances (HS) mainly derived from allochthonous origins and AOM characteristics was performed to hypothesize impacts of AOM transformation processes on the water quality of eutrophic water resources. Each AOM fraction underwent a specific evolution pattern, depending on its composition. Impacts of humification-like processes were predominant over release of biopolymers due to cells decay and led to an increase in the hydrophobic compounds part and molecular weights over time. However, the hydrophilic fraction remained the major fraction whatever the growth stage. Organic compounds generated by maturation of these precursors corresponded to large and aliphatic structures.

## 1. Introduction

Natural organic matter (NOM) is ubiquitous and its molecular structure is extremely complex. In aquatic environments, NOM is mainly derived from soils, terrestrial plants, aquatic organisms and human activities and its structure evolves continuously due to humification processes.

Humification results from various processes of degradation of organic material and rearrangement of the original products, involving biotic and abiotic reactions. These processes slowly lead to the formation of compounds refractory to biodegradation: the humic substances (HS). HS are composed of humins, humic acids (HA) and fulvic acids (FA), which are experimentally defined by pH conditions: humins are not soluble, HA are precipitated at pH 2 and FA are soluble whatever the pH [1]. HS have neither a constant composition nor a clearly identified structure [2]. It is now well stated that humification processes are very complex and several reaction pathways associated to NOM structural models were developed to explain HS genesis. Two main types of models were proposed: polymer models and molecular aggregates models.

HS genesis pathways by polymerization were described by Stevenson [3]. In the polymers models, NOM molecules are large polymers with a chemical structure different from their precursors linked with covalent bonds [4]. Polymer models include theories of lignin, polyphenols, tannins, abiotic condensation of sugars and amino-acids (Maillard type reaction) and plant derived humic substances [3,5,6,7].

In the aggregates models, NOM is composed of supramolecular aggregates of plant degradation products linked with non-covalent bonds (Van der Waals forces, π–π interactions, hydrophobic interactions and hydrogen bonds) [8,9]. Supramolecular aggregates are formed with individual components of low molecular weights—1 to 1.5 kDa—according to Piccolo, and Sutton and Sposito [8,9]. Evidence makes this theory currently the more adequate, with the relatively low molecular weights structures observed for HA (mainly in the range of 0.5 and 30 kDa according to several studies) [10,11,12].

Humification tends to increase complexity, aromatic carbon content [7,13] and apparent molecular weight of organic compounds [14]. An interesting approach to study these processes consists in fractionating organic matter according to physical (molecular weight fractionation) or chemical properties (fractionation according to the hydrophobic character by using polymeric XAD resins). The obtained fractions are linked to environmental processes and origin of the organic compounds. Organic material from allochthonous sources, such as plants decay from soil run off is mainly hydrophobic with high molecular weight; autochthonous contribution (phytoplankton and bacteria) is composed of more hydrophilic organic molecules of low molecular weight [15,16]. Organic matter produced by microalgae and cyanobacteria, thus, contains 57% to 71% of hydrophilic compounds according to recent works, depending on the growth phase and the species [17,18,19,20,21].

Humification processes and NOM structures were extensively studied in soils and in a lower extent in aquatic environments. Indeed, the previously described models were designed for NOM derived from soils, where plant contribution is predominant compared to microbial biomass (<5% of total carbon of soils according to Kassim *et al*. [22]). The chemical composition of organic matter derived from micro-organisms, which is different from that of plants, was thus not really taken into account. However, microbial activity can be a major component of NOM in natural waters and especially in the eutrophic ones, in which contribution of algal blooms cannot be neglected. Evolution of NOM in eutrophic waters is not well understood, although NOM tends to accumulate in these environments, which is a serious problem for drinking water production.

The objective of this work was to investigate how algal organic matter (AOM) evolves under specific bio-physico-chemical conditions during and after senescence of algae (*Euglena gracilis*) and cyanobacteria (*Microcystis aeruginosa*), in batch experiments. Specific UV absorbance (SUVA) index, as well as a combination of organic matter fractionations according to hydrophobicity and apparent molecular weight, were used to assess the evolution of the AOM characteristics from the freshly produced stage (stationary phase) to the degraded stage (advanced decline phase, after one year and four months of cultivation). A comparison with HS properties from soil and surface water resource allowed building hypotheses about AOM transformation pathways in the long term, and its possible consequences for the quality of eutrophic water resources.

## 2. Results and Discussion

### 2.1. Evolution of the Hydrophobic and Aromatic Characteristics

Table 1 shows the evolution of the hydrophobic and aromatic properties of AOM fractions and makes a comparison with NOM fractions.

**Table 1:** Comparison of aromatic (SUVA) and hydrophobic properties (organic matter fractions content relative to dissolved organic carbon (DOC)) of algal organic matter (AOM) at various growth stages with natural organic matter (NOM).

Growth Phase		Stationary Phase ^1^		Decline Phase ^1^		Advanced Decline Phase	
Origin	Fractions	% of DOC	SUVA ^2^	% of DOC	SUVA ^2^	% of DOC	SUVA ^2^
*Euglena gracilis*	Total	–	13.9 ± 0.1	–	11.2 ± 0.1	–	12.1 ± 0.1
	HPO ^3^	18	19.5 ± 1.6	24	26.4 ± 3.9	32	33.1 ± 0.9
	TPH ^4^	13	17.1 ± 1.4	15	13.5 ± 1.4	20	19.8 ± 1.1
	HPI ^5^	69	8.4 ± 0.6	61	6.9 ± 0.1	48	9.9 ± 0.1
*Microcystis aeruginosa*	Total	–	10.7 ± 1.3	–	10.4 ± 0.8	–	10.4 ± 0.1
	HPO ^3^	20	12.0 ± 0.4	24	19.0 ± 2.7	24	20.8 ± 0.4
	TPH ^4^	19	7.3 ± 0.6	29	4.0 ± 1.0	28	15.3 ± 0.3
	HPI ^5^	61	11.9 ± 0.5	47	11.6 ± 0.4	48	9.2 ± 0.8
**Origin**	**Fractions**	**% of DOC**			**SUVA ^2^**		
Pigeard pond	Total	–			36.5 ± 0.3		
	HPO ^3^	43			52.8 ± 0.8		
	TPH ^4^	21			27.2 ± 0.9		
	HPI ^5^	36			14.1 ± 0.1		
Suwannee River	HA	–			67.9 ± 0.5		
	FA	–			47.4 ± 0.5		
Peat	HA	–			101.0 ± 0.5		

^1^ data from Leloup *et al*. [21]; ^2^ Specific UV absorbance (SUVA) in L·cm^−1^·gC^−1^; ^3^ HPO: Hydrophobic fraction; ^4^ TPH: Transphilic fraction; and ^5^ HPI: Hydrophilic fraction.

Several authors already studied the evolution of AOM characteristics between the exponential and stationary growth phases. During this period AOM mainly derives from extracellular organic matter (EOM) release due to photosynthetic activity of living cells. Only little evolution was reported and no general conclusion can be drawn as regards HPO or HPI content increase according to the works by Henderson *et al*., Leloup *et al*., and Pivokonsky *et al*. [18,21,23].

**Figure 1:**
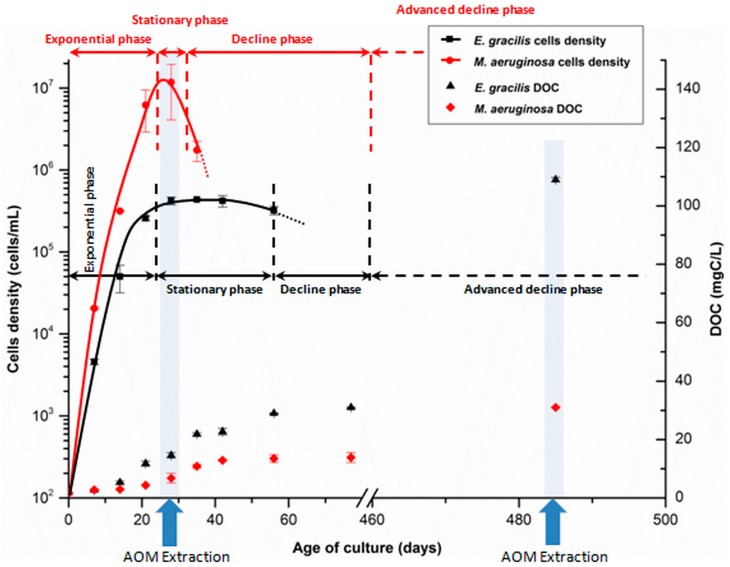
Growth curves and extraction time points of *E. gracilis* and *M. aeruginosa.* Cells densities were determined by flow cytometry in a previous study [21].

Highest intracellular organic matter (IOM) release rate is expected to occur between the stationary and decline phases because cells tend to massively die during the blooms collapse. This was especially the case for *M. aeruginosa* (Figure 1). Recent studies compared the hydrophobic characteristics of IOM and EOM [23,24]. They demonstrated that IOM is more hydrophilic than EOM (69%–74% for EOM *vs*. 87%–90% for IOM). A slight increase in the Hydrophilic fraction (HPI) part (as well as the corresponding hydrophobic fraction (HPO) percentage decrease) would thus be expected from cells decay. However, contrary to what was expected, a significant increase in the HPO and transphilic fraction (TPH) parts was observed while the HPI percentage decreased for both species (−8% to −14%). This evolution continued until the end of the experiment, after more than a year of cultivation but stage of HA production was not reached (<3% of DOC). Several processes could explain these unexpected modifications. After the cells death, two additional processes—photo-induced reactions (including dissolution of cells debris) and leaching of particulate organic matter—could contribute to the HPO and TPH increase, as well as transformation of organic compounds (especially the HPI fraction, which is known to be the most labile [25] by microbial activity. It cannot be neglected anymore when compared to exponential and stationary growth phases, during which biological activity form phytoplankton was predominant. Impacts of these processes thus overcame the expected HPI increase from IOM release by cells decay. These processes were then predominant and their consequences on AOM properties—increase in HPO and TPH parts—were consistent with the humification theories.

Some differences can be observed between the two species. Much more evolution of the organic matter properties (aromaticity and hydrophobicity) was observed during the first four months of cultivation than during the following year in the case of *M. aeruginosa*, compared to *E. gracilis*. An equilibrium state (according to DOC content) was reached after four months (decline phase) for the cyanobacteria, when the same state of transformation was only achieved during the advanced decline phase for the alga (after more than 12 months). The faster evolution of *M. aeruginosa*’s AOM was probably due to a shorter stationary phase (only seven days long for the cyanobacteria *vs.* 34 days for the alga, Figure 1), which allowed degradation and transformation processes from biotic and abiotic factors to occur earlier. *E. gracilis* population declined slowly, likely because of mixotrophy.

Despite the impacts of transformation processes, AOM remained mainly hydrophilic (48% of DOC for both species) a long time after senescence, contrary to NOM from natural waters (36% of DOC for Pigeard pond). Humification-like processes affecting AOM may be different from those occurring in soils (probably because the characteristics of precursors are different and the resulting humic-like substances characteristics may also be different) and/or may require a long time to make AOM characteristics tend to the level of organic matter from allochthonous origin. However, one limitation of this study is that it does not reflect seasonal variations of light intensity and temperature occurring in natural systems over the year, so the impact of transformation processes on the AOM characteristics is probably underestimated.

Whatever the growth phase, SUVA indexes of AOM were lower than those of NOM from allochthonous origin (Table 1), which is consistent with results from Weishaar *et al*. [26] and correlates with its high HPI content. Leloup *et al*. [21] already discussed the evolution of AOM SUVA between the stationary and decline phases and compared SUVA values between *E. gracilis* and *M. aeruginosa*. From the stationary phase to the advanced decline phase, SUVA index of HPO from both species increased. SUVA of TPH and in a lesser extent of HPI decreased between the stationary and decline phases and increased between the decline and advanced decline phases, except for HPI of *M. aeruginosa*,which did not evolve so much*.* Transformation processes affecting HPO seem to be different from the TPH and HPI fractions, probably because the latter are known to be more labile and subject to biodegradation than HPO [25]. The HPO fraction seemed to undergo a slow one-step process, which started immediately after the stationary phase, while the HPI and TPH fractions were affected by a two-step process. Thus, in a first time, both fractions may be degraded by bacterial activity into less aromatic compounds between the stationary and decline phases. In a second step, they may be condensed and reassembled into more complex and aromatic structures.

**Figure 2:**
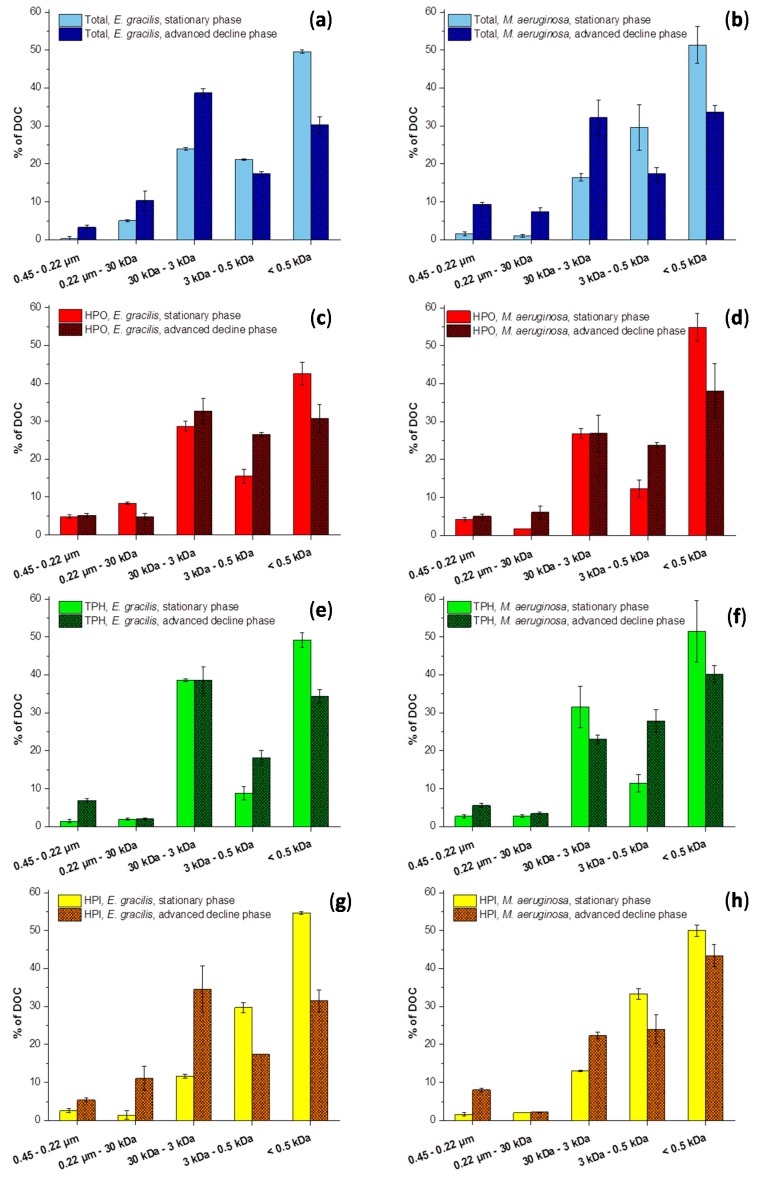
Comparison of size fractionation of dissolved organic matter: Evolution between the stationary and advanced decline phases: (**a**,**b**) are total samples from *E. gracilis* and *M. aeruginosa*; (**c**,**e**,**g**) are, respectively, HPO, TPH and HPI fractions of AOM produced by *E. gracilis* and (**d**,**f**,**h**) are, respectively, HPO, TPH and HPI fractions from *M. aeruginosa*.

### 2.2. Size Fractionation of Organic Matters in Different Stages

A size fractionation protocol was applied in order to separate organic matter in several fractions according to the apparent molecular weight of molecules. Such a protocol well correlates with the fractionation according to the hydrophobic character and was used here both to evaluate the repartition of the size of organic molecules during the different growth phases of the cultures of *E. gracilis* and *M. aeruginosa* and to identify the most abundant ones in the HA, FA, HPO, TPH and HPI fractions from different origins.

**Figure 3:**
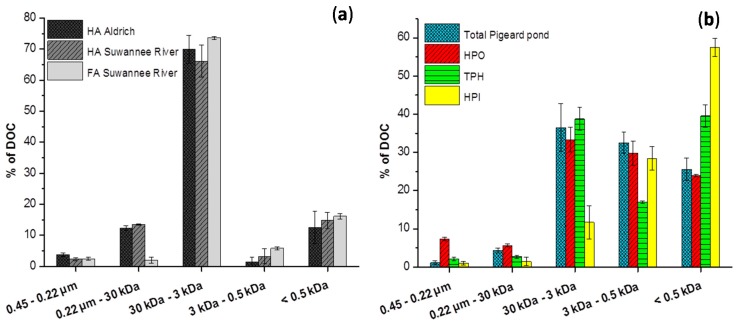
Size fractionation of HA and FA from peat and the Suwannee river are given in (**a**) and that from Pigeard pond in (**b**).

Figure 2a–h show size fractionation of total samples from cultures, HPO, TPH and HPI fractions during stationary phase (fresh organic material) and advanced decline phase (degraded material). Figure 3a,b show size fractionation of organic material from the Suwannee river and peat (a), as well as that of fractions from the Pigeard pond (b) for comparison.

Molecular size distribution was dependant on the species and the growth phase but a similar evolution was noticed for both species. Size distribution of AOM was modified and molecular weights tended to increase between the stationary and advanced decline phases. During the stationary phase and whatever the HPO, TPH and HPI fractions, molecules less than 0.5 kDa accounted for the major size fraction of freshly produced AOM. Results of total samples were consistent with those obtained by Henderson *et al*. [18], who observed that 30%–80% of AOM is less than 1 kDa. Then, after more than one year of evolution, the part of size fraction more than 0.5 kDa increased. This phenomenon can be explained by combination of biopolymers release from cells death as IOM is of larger size than EOM [20] and transformation processes of AOM over a long period. However, the impacts of transformation processes were predominant, as previously explained from the observed HPO increase. Increase in molecular size is, thus, in accordance with consequences of humification-like processes [14] and reflects the advance of transformation of AOM.

Specific evolution pathways were observed for HPO, TPH and HPI fractions but similar tendencies were noticed for both species. For the HPO (Figure 2c,d) and TPH (Figure 2e,f) fractions, the part of size fraction between 3 and 0.5 kDa increased while the one less than 0.5 kDa was reduced. On the contrary, part of the size fraction of hydrophilic compounds (Figure 2g,h) comprised between 30 and 3 kDa increased and those of fractions less than 3 kDa decreased. Because HPI remains predominant in AOM, it imposed the evolution of total samples (Figure 2a,b). Labanowski and Feuillade observed in natural waters that the greater the size, the higher the aromaticity [25]. However, during advanced decline phase, HPI fraction had similar content of 3–30 kDa size fraction but lower SUVA index than the HPO fraction. Thus, HPI produced by maturation of AOM contained large and aliphatic structures, while HPO structures were much more aromatic. As regards AOM, some studies provided evidences that the main part of carbohydrates is contained in the HPI fraction (50%–80%), while 60%–65% of proteins are contained into the HPO fraction [18,27]. TPH fraction only contains between 5%–15% of the carbohydrates and proteins according to the authors previously cited. Biochemical composition of HPO and TPH fractions coming from the growth of *E. gracilis* and *M. aeruginosa* was analyzed by mass spectrometry.

The results were different during the early stationary phase: proteic markers were relatively more abundant in HPO than in TPH, contrary to the sterols ones [28]. These differences in composition may explain why transformation and stabilization processes were different for each fraction. Humification-like processes involving polymerization of polysaccharides may be predominant for HPI, leading to an increase in the 3–30 kDa size fraction part and explaining the obtained large and aliphatic structures. For HPO and TPH fractions, transformation processes involving condensation of proteins (more aromatic than polysaccharides) may rather be predominant and lead to an increase in the part of size fraction 0.5–3 kDa.

Chemical composition of plants and algae are very different so theories commonly used to explain formation of HS in soils cannot be directly applied to explain AOM transformation. Lignin, tannin and polyphenols humification theories do not apply to AOM because they are minor constituents of algae and microorganisms cells [29]. However, polyphenols theory may apply indirectly. Indeed, this theory could be envisaged after AOM bacterial assimilation because polyphenols can be synthesized from organic carbon sources by micro-organisms [3]. Polyphenols can be degraded into quinones and reactions can occur spontaneously with sugars or amino-acids for example. Because bacteria degrade preferentially labile organic compounds, such as those contained in the HPI and TPH fractions, the first steps of polyphenols theory may explain the evolution of these two fractions, whereas reactions between by-products and other organic compounds may affect each of the three fractions. Cyanobacteria and green algae are known to produce significant levels of terpenes [30], which are stored or released during stationary phase or senescence, depending on the species and the environmental conditions [31]. Thus, terpenes association with proteins and polysaccharides could also be envisaged.

Composition of AOM varies depending on the species, the growth phase and the environmental conditions [32]. Polysaccharides, proteins (30% to 55% of dry algal biomass according to López *et al*. [33]), peptides, amino acids and other organic acids like fatty acids are the main constituents [34]. Condensation and polymerization reactions of fatty acids or poly-unsaturated lipids can also be envisaged. However, Maillard type reactions leading to condensation between polysaccharides and amino-acids are unfavorable or very slow under the used conditions. Indeed, they need to be catalyzed by temperature or, like in soils by manganese or iron oxides according to Jokic* et al*., and *Qi et al*. for example [35,36].

**Figure 4:**
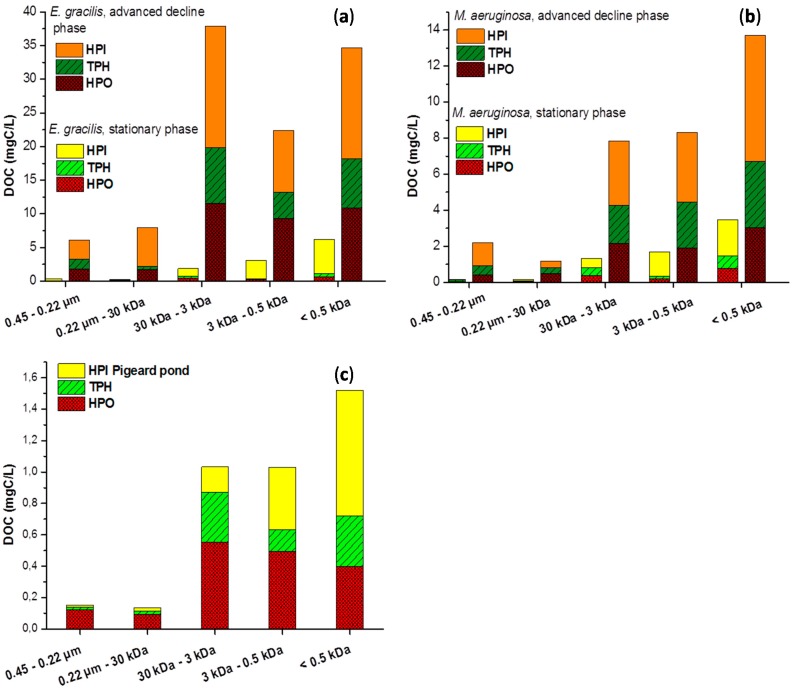
Concentration of HPO, TPH and HPI fractions in the different size fractions during the stationary and advanced decline phases for *E. gracilis* (**a**) and *M. aeruginosa* (**b**) and during winter for Pigeard pond (**c**).

Figure 3a,b compare the size fractionation of organic matter derived from soil and surface water resources and show that size fraction between 30 and 3 kDa was largely predominant (>65%) in HA and FA. High content in organic molecules of high molecular weight correlates with an advanced degree of humification [14]. Thus, humification processes of allochthonous organic material mainly led to the formation of 3–30 kDa compounds. Some consequences of eutrophication and AOM humification-like processes can be anticipated for natural organic matter from surface water resources. HPO and FA fractions are experimentally similar, also when comparing FA from the Suwannee River and HPO extracted from phytoplankton during the advanced decline phase, 3–30 kDa content was significantly lower for the latter. This difference can be explained either by the presence of different precursors leading to the formation of different HS or by requirement of a very long time to achieve a second stage of evolution for AOM, during which 3–30 kDa part of the HPO would increase. Nevertheless, both assumptions result in a low content of size fraction 3–30 kDa for HPO from the eutrophic Pigeard pond (Figure 3a), compared to the river not affected by blooms (37% *vs*. 75%). This gap is expected to widen in the long term because of annual AOM inputs and consequences of its humification-like processes. Then, another impact of eutrophication is the recurrent input of freshly produced HPI by phytoplankton. Although sampling was performed during winter, when phytoplanktonic activity was weak, the size profile of the HPI fraction from Pigeard pond was similar to those of fresh AOM, in which fractions lower than 3 kDa were the most abundant and especially the size fraction less than 0.5 kDa. This phenomenon cannot result from an artifact of the fractionation procedure because the HPI size profile was significantly modified during the advanced decline phase, especially for *E. gracilis*. It rather seems to indicate that the degree of transformation of HPI remains low in Pigeard pond. Because HPI content is expected to increase in eutrophic water resources in the long term, contribution of hydrophilic compounds in each size fraction (Figure 4) is expected to increase in the affected water resources. The extent of the consequences of eutrophication on the characteristics of NOM is likely to depend on the humification-like processes of AOM.

## 3. Experimental Section

AOM was extracted from mono-specific cultures of algae and cyanobacteria. Characteristics of AOM were compared with those of organic matter extracted during winter from a shallow artificial and eutrophic pond located in the Limousin area (latitude 45°54ʹN and longitude 1°11ʹE, Nieul, France), the Pigeard pond. This pond is well known for its high algal productivity. A characterization of organic matter fractions coming from Pigeard pond, HA provided by Aldrich (Saint-Louis, MO, USA) and HA (2S101H) and FA (2S101F) extracted from the Suwannee River and provided by the International Humic Substances Society (IHSS, Denver, CO, USA), was performed for comparison.

### 3.1. Samples Preparation

#### 3.1.1. Algae and Cyanobacteria Cultivation

Non axenic strains of *E. gracilis* and *M. aeruginosa* provided by the National Museum of Natural History (Paris, France), were used to stand for microbial activity happening in natural waters. The two species were grown in 1 L Erlenmeyer flasks filled up with 500 mL of synthetic sterilized Chu 10 modified medium, according to the protocol used by Leloup *et al*. [21]. Cultures were grown at 23 °C with a 15 h/9 h light/dark cycle under 30 µmol·photon·m^−2^·s^−1^ illumination. The same conditions were maintained until the end of experiment. Progressive evaporation occurring during the experiment was taken into account by application of a correcting factor for calculations, depending on the measured final volume on initial volume ratio.

#### 3.1.2. Organic Matter Extraction Procedure

AOM was extracted from cultures during the stationary growth phase (21 and 28 days for *E. gracilis* and *M. aeruginosa*, Figure 1) and the advanced decline phase, after one year and four months of cultivation. The duration of the experiment (more than a year) was chosen to reflect the transformation of AOM over a long period of time after its production and because fresh AOM inputs occur on an annually basis in eutrophic water resources. Both AOM and NOM from Pigeard pond were extracted according to the Malcolm and MacCarthy’s protocol [37], by adsorption on DAX-8 and XAD-4 resins in series. The fractionation protocol allowed separating organic matter into four fractions: HA, HPO, TPH and HPI fractions. However as HA were not produced in sufficient quantity for analyses (<3% of DOC), only three fractions were characterized in this work. The partition coefficient *kʹ* was fixed to 50.

Cultures were first centrifuged at 6000× *g* during 20 min at 4 °C. Natural samples and centrifuged culture medium were both filtered on 0.45 µm cellulose nitrate membrane and HA were removed by samples acidification at pH 2 with HCl 37% and filtration on 0.45 µm. HPO were retained on DAX-8 resin; TPH were retained on XAD-4 resin and HPI were not retained on resins and remained in the sample with salts. Before desorption, resins were rinsed with formic acid at pH 2. HPO and TPH fractions were desorbed with Acetonitrile 75%/Water 25% (*v*/*v*). Organic matter recovery is almost 100% by using such mixture as the eluent [38]. Acetonitrile was finally removed by evaporation and both fractions were freeze-dried.

### 3.2. Organic Matter Characterization

#### 3.2.1. Size Fractionation

pH of each sample was adjusted to 6.5 prior to size fractionation, which was performed by ultrafiltration using a diafiltration method. This method consists in keeping the volume of sample constant in the ultrafiltration cell by adding a sodium nitrate solution with the same ionic strength as the sample. It allows reducing measurements disturbance due to polarization of concentration phenomenon. The detailed protocol is described elsewhere [25]. Ionic strength was calculated from the electrical conductivity of the sample (measured with a WTW LF 538 conductimeter of precision ± 0.5%) by using the Marion–Babcock Equation (Equation (1)) [39].

log *I* = 1.159 + 1.009logχ; *I* ionic strength in mmol·L^−1^ and χ conductivity in mS·cm^−1^ (1)

Organic molecules were separated into five fractions (four fractions comprised between: 0.45 and 0.2 µm, 0.2 µm and 30 kDa, 30 and 3 kDa, 3 and 0.5 kDa, and one fraction less than 0.5 kDa) by successive filtration of sample through 0.45 and 0.2 µm cellulose nitrate membranes, YM Amicon membrane of 30 and 3 kDa, as well as YC Amicon membrane of 0.5 kDa molecular weight cut-offs. The samples filtration was carried out under 3 bars pressure of compressed air. The proportion of each fraction was determined by measuring DOC content of the filtrates and the retentates. Analyses were performed in duplicates.

#### 3.2.2. Dissolved Organic Carbon Measurements

DOC measurements were performed by a Shimadzu TOC-L analyzer (precision ± 2%, Shimadzu, Kyoto, Japan) according to the Non-Purgeable Organic Carbon measurement procedure.

#### 3.2.3. Specific UV Absorbance Index

The SUVA index is defined as the ratio of the UV absorbance at 254 nm to the DOC concentration. The UV absorbance at 254 nm was measured by using a Shimadzu PharmaSpec 1700 spectrophotometer (precision ± 0.005 cm^−1^; Shimadzu, Kyoto, Japan) with 1 cm-long quartz cells. This index allows estimating the aromaticity of each fraction and correlates with the hydrophobicity of organic molecules [26,38]. Analyses were performed in duplicate.

## 4. Conclusions

The evolution of AOM properties in the long term predominantly resulted from humification-like processes than from a single process of biopolymer release from cells decay. Compounds formed by AOM transformation were either different from HS generated by humification because the precursors were different or required a very long time to allow their properties to become closer. Each organic fraction (HPO, TPH and HPI) evolved differently from the others, depending on its composition, but these processes were similar for both the algae and the cyanobacteria. A faster evolution of *M. aeruginosa*’s AOM compared to that of *E. gracilis* was probably due to a shorter stationary phase, which allowed degradation and transformation processes from biotic and abiotic factors to occur earlier. *E. gracilis* population declined slowly, likely because of mixotrophy. The extent of the consequences of eutrophication on the NOM characteristics of natural water resources is likely to depend on the impacts of humification-like processes of AOM: increase in HPI content whose properties would remain close to freshly produced HPI fraction of phytoplanktonic origin associated to a decrease in molecular weights of HPO compounds. Further *in situ* studies would be required to confirm these assumptions.
